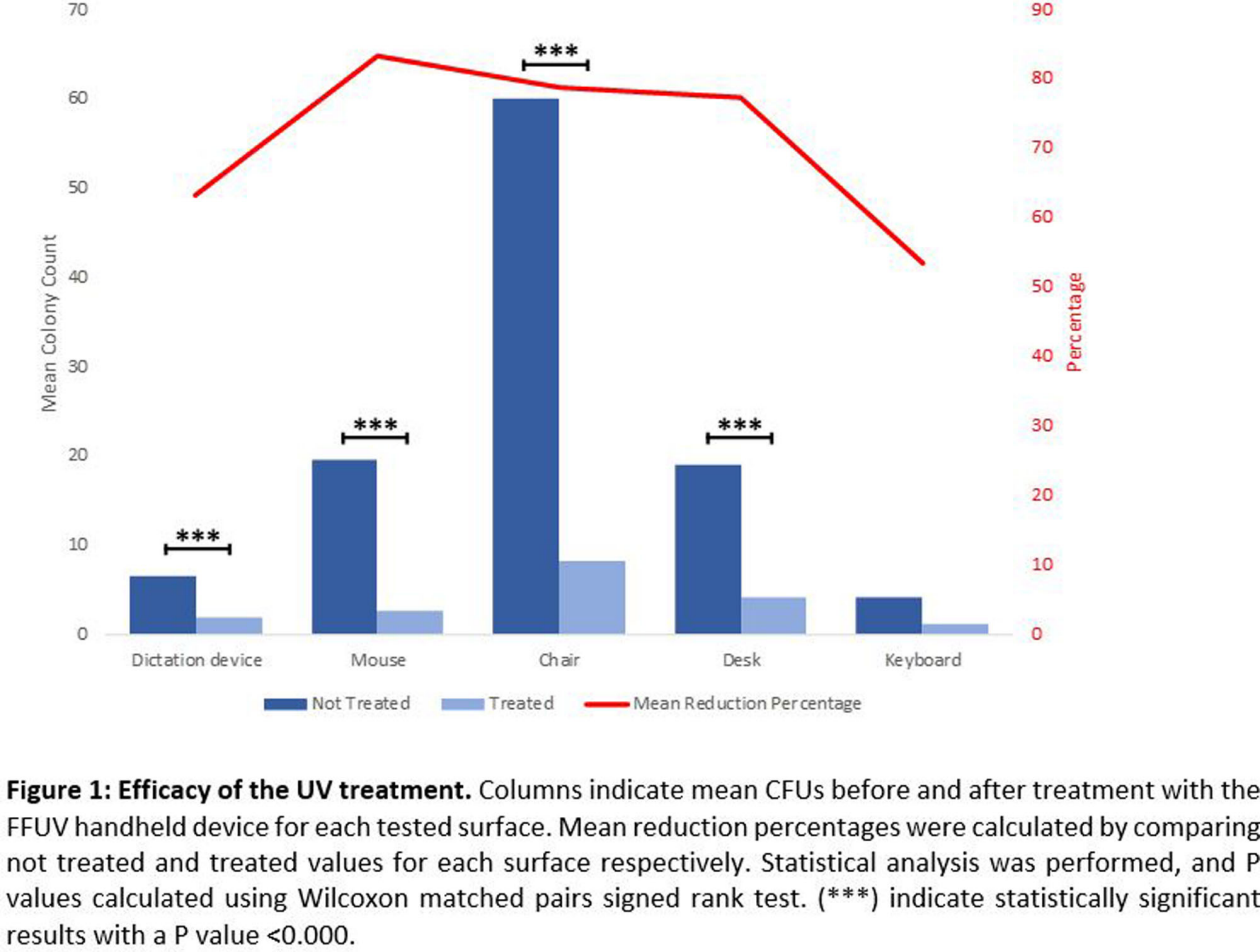# Filtered handheld far-ultraviolet disinfection device in reducing environmental pathogens from high-touch clinical surfaces

**DOI:** 10.1017/ash.2024.242

**Published:** 2024-09-16

**Authors:** Layale Yaghi, Rita Wilson-Dib, Sherry Cantu, Piyali Chatterjee, Chetan Jinadatha, Roy Chemaly, Amy Spallone

**Affiliations:** The University of Texas MD Anderson Cancer Center; MD Anderson Cancer Center; Central Texas Veterans Health Care System

## Abstract

**Background:** Healthcare-acquired infections (HAIs) continue to be a major challenge. In fact, an increased risk of HAIs has been linked to high-touch surfaces contaminated with multidrug-resistant organisms (MDROs), and enhanced environmental disinfection is linked to reduced HAI rates. Recently, more focus has been placed on emerging disinfection technologies, such as UV light-producing portable device that emits light at a wavelength of 222 nm, which has previously demonstrated germicidal capabilities at short contact times. In this study, we aim i) to evaluate the efficacy of a filtered far-UV-C handheld device (FFUHH) to reduce bacterial loads on high-touch surfaces in clinical workrooms in a cancer center, and ii) to isolate, identify and establish a genetic relationship between these environmental clinically significant pathogens and the ones recovered from patients. **Methods:** Samples were collected weekly on a rotating schedule over a 24-week period from five high-touch items (dictation device, mouse, armchair, desk, and keyboard) in multiple clinical work rooms on hematologic malignancy and stem cell transplant units. Contact plates for colony count and swabs were collected pre- and post-intervention with the FFUHH on standardized adjacent areas respectively for each surface. The swabs were enriched and cultured on selective media to isolate clinically significant pathogens. Whole genome sequencing (WGS) was then performed on environmental pathogens validated by MALDI-TOF as well as clinical samples collected from patients in the same unit around the time of environmental sample collection. **Results:** A total of 440 plates, 220 pre- and 220 post-interventions, were collected and analyzed. The highest mean colony count pre-treatment was detected from the armchairs and the lowest for the keyboards. The mean reduction of colony forming units (CFUs) ranged between 53% for the keyboard and 83% for the mouse. The reduction was statistically significant across all surfaces with P values < 0.05, except for the keyboard (Figure 1). We isolated many pathogens of the human microbiota identified by MALDI-TOF such as Micrococcus luteus, S. capitis as well as methicillin-resistant S. epidermidis, S. haemolyticus and S. hominis. We also identified several Candida parapsilosis, Pseudomonas stutzeri, one Listeria grayi and one Acinetobacter baumanni. Finally, WSG allowed us to further characterize an environmental multi-drug resistant S. epidermidis ST5 strain associated with patient bacteremia, and ST16 strains detected on surfaces both pre- and post-FFUHH treatment. **Conclusion:** The FFUHH effectively reduced the microbial burden on high-touch surfaces in clinical workrooms on hematologic malignancy and stem cell units.

**Figure:**